# Relationships between Parental Education and Overweight with Childhood Overweight and Physical Activity in 9–11 Year Old Children: Results from a 12-Country Study

**DOI:** 10.1371/journal.pone.0147746

**Published:** 2016-08-24

**Authors:** Stella K. Muthuri, Vincent O. Onywera, Mark S. Tremblay, Stephanie T. Broyles, Jean-Philippe Chaput, Mikael Fogelholm, Gang Hu, Rebecca Kuriyan, Anura Kurpad, Estelle V. Lambert, Carol Maher, José Maia, Victor Matsudo, Timothy Olds, Olga L. Sarmiento, Martyn Standage, Catrine Tudor-Locke, Pei Zhao, Timothy S. Church, Peter T. Katzmarzyk, ISCOLE Research Group

**Affiliations:** 1 African Population and Health Research Center, P.O. Box 10787–00100, Nairobi, Kenya; 2 Department of Recreation Management and Exercise Science, Kenyatta University, P. O. Box 43844, Nairobi, 00100, Kenya; 3 Children’s Hospital of Eastern Ontario Research Institute, 401 Smyth Road, Ottawa, ON K1H 8L1, Canada; 4 Pennington Biomedical Research Center, 6400 Perkins Road, Baton Rouge, LA 70808–4124, United States of America; 5 Department of Food and Environmental Sciences, University of Helsinki, P. O. Box 3, 00014, Helsinki, Finland; 6 St. Johns Research Institute, 100 Feet Rd, John Nagar, Koramangala, Bengaluru, Karnataka 560034, Bangalore, India; 7 Division of Exercise Science and Sports Medicine, Faculty of Health Sciences, University of Cape Town, Anzio Road, Cape Town, 7935, South Africa; 8 Alliance for Research in Exercise Nutrition and Activity (ARENA), School of Health Sciences, University of South Australia, North Terrace, Adelaide SA 5001, Australia; 9 CIFI2D, Faculdade de Desporto, University of Porto, Rua Dr. Plácido Costa, 91–4200.450, Porto, Portugal; 10 Centro de Estudos do Laboratório de Aptidão Física de São Caetano do Sul (CELAFISCS), R. Heloísa Pamplona, 269—Fundação, São Caetano do Sul—Sao Paulo, 09520–320, Brazil; 11 School of Medicine, Universidad de los Andes, Carrera Primera 18A-12, Bogotá, Colombia; 12 Department for Health, University of Bath, Claverton Down, Bath, BA2 7AY, United Kingdom; 13 Department of Kinesiology, University of Massachusetts Amherst, 30 Eastman Lane, Amherst, MA 01003–9258, United States of America; 14 Tianjin Women’s and Children’s Health Center, 96 Guizhou Road, Heping, Tianjin, China; National Eye Institute, UNITED STATES

## Abstract

**Background:**

Globally, the high prevalence of overweight and low levels of physical activity among children has serious implications for morbidity and premature mortality in adulthood. Various parental factors are associated with childhood overweight and physical activity. The objective of this paper was to investigate relationships between parental education or overweight, and (i) child overweight, (ii) child physical activity, and (iii) explore household coexistence of overweight, in a large international sample.

**Methods:**

Data were collected from 4752 children (9–11 years) as part of the International Study of Childhood Obesity, Lifestyle and the Environment in 12 countries around the world. Physical activity of participating children was assessed by accelerometry, and body weight directly measured. Questionnaires were used to collect parents’ education level, weight, and height.

**Results:**

Maternal and paternal overweight were positively associated with child overweight. Higher household coexistence of parent-child overweight was observed among overweight children compared to the total sample. There was a positive relationship between maternal education and child overweight in Colombia 1.90 (1.23–2.94) [*odds ratio (confidence interval)*] and Kenya 4.80 (2.21–10.43), and a negative relationship between paternal education and child overweight in Brazil 0.55 (0.33–0.92) and the USA 0.54 (0.33–0.88). Maternal education was negatively associated with children meeting physical activity guidelines in Colombia 0.53 (0.33–0.85), Kenya 0.35 (0.19–0.63), and Portugal 0.54 (0.31–0.96).

**Conclusions:**

Results are aligned with previous studies showing positive associations between parental and child overweight in all countries, and positive relationships between parental education and child overweight or negative associations between parental education and child physical activity in lower economic status countries. Relationships between maternal and paternal education and child weight status and physical activity appear to be related to the developmental stage of different countries. Given these varied relationships, it is crucial to further explore familial factors when investigating child overweight and physical activity.

## Introduction

Maintaining a healthy body weight and participating in adequate levels of physical activity have the potential to improve cardiorespiratory, muscular, bone, and psychosocial health, while reducing the risk for several non-communicable diseases (NCDs) [1–3]. The health risks associated with overweight or obesity and inadequate physical activity levels are a particular problem among children owing to increased risk for morbidity and premature mortality in their adult years [4, 5, 6]. Childhood overweight and obesity is a serious public health challenge, even in low- and middle-income countries [7]. Further, it is recommended that children and youth aged 5 to 17 years accumulate at least 60 minutes of moderate-to-vigorous intensity physical activity (MVPA) daily in order to accrue positive health benefits [8]. Evidence has shown that parents play a crucial role in shaping their children's health behaviors, and parental education level and weight status have been associated with these outcomes in children [9–25]. However, discrepancies and inconsistencies in the existence, direction, and effect size of these relationships across different studies have been attributed to differences in study design, sampling, sociodemographic factors, types of accelerometers, or cut points used to categorize MVPA [9].

Evidence from several upper-middle and high income countries (Australia, Croatia, Germany, Italy, Mexico, and the USA) has shown that parental overweight, and more predominantly maternal overweight status, is a risk factor for childhood overweight [10–18]. Fewer studies have investigated associations between parental education level and childhood overweight status. One study on Latinos in the USA found that mothers with higher Spanish language scores had children with higher BMI compared to mothers with lower language scores [9]. Fathers’ education was also positively correlated with childhood obesity in Croatia [19]. Longitudinal data from the USA showed a negative relationship between childhood obesity and education level of the household head; however, this relationship was not consistent across race and ethnic groups [20]. Similarly, Danish children were more likely to become overweight if their parents were less educated [21].

The same study of Latinos in the USA found that higher maternal BMI was associated with lower child MVPA, reflective of shared family lifestyles, behaviour, and environments [9]. Maternal education was also negatively associated with child MVPA [9]. Systematic review evidence from sub-Saharan African countries showed higher parental education level and BMI (both strongly and positively correlated with higher socioeconomic status; SES), to be negatively associated with lower child physical activity and higher adiposity [22, 23]. However, a different systematic review encompassing 150 studies mainly from North American, European, and a few Oceanian countries found that mother’s education level was consistently positively correlated with physical activity among adolescents (12–18 years) but not children (3–12 years) [24]. With the latter in mind, other work has shown parent education not to be a significant predictor of physical activity levels among young children [25].

In light of these disparate results, which may be related to the developmental stage of different countries, the objective of this paper was to investigate relationships between reported maternal and paternal education level or overweight, and (i) directly measured child overweight, (ii) meeting physical activity guidelines, and (iii) explore household coexistence of parental and children overweight, among children (9–11 years). The analysis would use data collected following a standard protocol in 12 countries across the world, varying widely in economic, human development, geographic, and social factors. This paper singles out two parental factors (education level and overweight) and examines their influence on childhood overweight and physical activity, rather than considering these parental factors as part of broader socioeconomic factors, while also allowing for contrasting of mother versus father education (or overweight) as correlates for childhood overweight and physical activity.

## Methods

### The ISCOLE project

The International Study of Childhood Obesity, Lifestyle and the Environment (ISCOLE) was designed to investigate the influence of behavioural settings and the physical, social, and policy environments on the observed relationships between lifestyle and weight status among school-aged children from 12 countries around the world [26]. While previous multi-country childhood obesity studies have focused on specific geographic regions (e.g. Europe), ISCOLE was designed to have global representation by including study sites from within high-, middle-, and low-income countries. Consequently, the 12 ISCOLE countries represent five major geographic regions of the world including Africa (Kenya, South Africa), the Americas (Brazil, Colombia, Canada, USA), Europe (Finland, Portugal, United Kingdom (UK)), South Asia (India), and the Western Pacific (China, Australia), with varying economic ranks [27]. The study design also incorporated comprehensive and robust indicators of lifestyle behaviours (e.g. physical activity, sedentary behaviour, food consumption, and sleep) and directly measured adiposity and physical activity. A detailed description of the ISCOLE protocol is published elsewhere [26]. Overall coordination, training in data collection methods, and data management for the ISCOLE project, including final quality control checks, was provided by the ISCOLE Coordinating Center staff at the Pennington Biomedical Research Center (Baton Rouge, USA). Data collection was conducted following ethical approval from the University of South Australia Human Research Ethics Committee (Australia), the Research Ethics Committee of the Municipal Health of Sao Caetano do Sul—Prima (Brazil), the Children’s Hospital of Eastern Ontario Research Ethics Board (Canada), the Biomedical Ethics Committee of Tianjin Women’s and Children’s Health Center (China), the Universidad de los Andes Committee on Research Ethics (Colombia), the Ethics Committee of the Hospital District of Helsinki and Uusimaa (Finland), the St. John’s Medical College and Hospital Institutional Ethical Review Board (India), the Kenyatta University Ethics Review Committee (Kenya), the Ethics Committee University of Porto (Portugal), the University of Cape Town Health Sciences Faculty Human Research Ethics Committee (South Africa), the University of Bath Research Ethics Committee for Health (UK), and the Pennington Biomedical Research Center Institutional Review Board for Research with Human Subjects (USA). Written informed consent and assent from all parents/guardians and participating children was also obtained.

### ISCOLE design

Recruitment targeted a sex-balanced sample of approximately 500 children 9–11 years of age from each site. The primary sampling frame in all sites was schools in urban and suburban areas, stratified by indicators of SES status in order to maximize variability within sites. The secondary sampling frame was classrooms having children with minimum variability around 10 years of age. ISCOLE data collection was conducted during a full school year, which varied across countries; however, data collection in all sites was conducted between September 2011 and December 2013. Any variations in recruitment strategies employed in the different countries are reported elsewhere [26].

### Anthropometry

Standing height was measured by trained research staff using a Seca 213 portable stadiometer (Birmingham, UK), with the participant as erect as possible and head positioned in the Frankfort horizontal plane. Weight was then measured using a portable Tanita Body Composition Analyser (Illinois, USA), after all outer clothing, heavy pocket items, shoes, and socks were removed. Body mass index (BMI) was derived from weight and height (kg/m^2^), and children were categorised based on the World Health Organization (WHO) BMI growth reference classification of thinness (z-score < -2); healthy weight (≥ -2 and ≤ +1); overweight (>+1 and ≤ +2); and obese (>+2) [28]. For the purpose of this study, the term “overweight” is used, and is inclusive of children classified as obese.

### Accelerometry

Physical activity was objectively measured using ActiGraph GT3X+ accelerometers (Florida, USA). Accelerometers were attached firmly to belts and worn at the waist on the right mid-axillary line. Participating children were instructed to wear the devices 24 hours per day for at least 7 consecutive days (at all times except when bathing or swimming), including an initial familiarisation day, maximizing the number of children providing at least 4 days of waking-hours wear time of ≥10 hours/day, including at least one valid weekend day. Time spent sleeping at night was first identified using a published algorithm, and non-wear time within the awake portion of the day was classified as 20 consecutive minutes of ‘0’ counts [29, 30]. Data from the accelerometers were downloaded, reviewed for completeness, and data reduction conducted using cut-points validated in children and youth [31]. MVPA was defined as ≥ 574 counts per 15 seconds [31]. Children meeting the WHO physical activity guideline of ≥ 60 minutes of daily MVPA [8], based on their mean daily minutes in MVPA, were also identified.

### Questionnaires

A Demographic and Family History Questionnaire was completed by one or both parent(s) or guardian(s) of the participating child, but collected information on the child’s biological parents. This questionnaire collected information on basic demographics, family health, and socioeconomic factors [26]. ISCOLE’s questionnaires were a compilation of several validated items obtained from existing questionnaires, and where no suitable alternatives were found, new questions were designed by content experts from the ISCOLE investigator team. To maximize the collection of valid and reliable data, technicians were trained on questionnaire administration in a standardised manner, and provisions were made to administer the questionnaire via an interview for participants with low literacy levels. Parental education was categorised as some college/associate degree, bachelor, or post-graduate degree (higher education level), compared to the remainder reporting that they completed high school, had some high school, or less than high school (lower education level). Parents were also categorised based on self-reported height and weight into underweight (< 18.5 kg/m^2^), normal weight (18.5–24.9 kg/m^2^), overweight (25.0–29.9 kg/m^2^), or obese (≥ 30.0 kg/m^2^) [32]. For the purpose of this study, the term “overweight” is inclusive of those classified as obese by these standards.

### Missing data

A total of 7372 children participated in ISCOLE. However, children who were missing information on BMI, physical activity, or mother’s and/or father’s education level, weight, and/or height, were excluded from these analyses. This resulted in a decrease to an analytic sample of 4752 (64.5%). The largest contribution to the missing data was father’s BMI; however, this was not country specific. The Fisher’s exact test was used to investigate whether there was a statistical difference between excluded children and those in the analytic sample. We found no differences in key variables including sex (*p* = 0.77), overweight status (*p* = 0.16), or meeting MVPA guidelines (*p* = 0.17).

### Statistical analysis

The statistical analyses presented in this paper were conducted using SAS version 9.3 (SAS Institute Incorporated, Cary, North Carolina, USA). Descriptive statistics (e.g. frequencies, means, and standard deviations) were calculated. The correlates included were maternal education, paternal education, maternal overweight and paternal overweight. Sex was included as a covariate in the final models. Multilevel modeling analysis was used to obtain parameter estimates for the associations between childhood overweight or meeting the WHO MVPA guidelines and parental education level or parental overweight status. This analytical method allows for the simultaneous examination of the effects at the individual (child), school, and country (site) levels. That is, it accounts for the hierarchical nature of the data. Multilevel general linear models (PROC GLIMMIX), including site as a fixed effect, were used to determine correlates of childhood overweight or meeting MVPA guidelines.

## Results

*Table 1* shows site-specific characteristics at the child and parental levels for 4752 children. Sites in Portugal, Brazil, and China had the highest proportion of children classified as overweight or obese (overweight), ranging between 46.9% and 42.2%, while the lowest proportion (18.8%) was in the Kenyan site. Across all sites, children were spending a mean of 45.4 to 71.2 minutes/day in MVPA, with as few as 16.2% of children from China, and as many as 64.0% of children from Finland, meeting MVPA guidelines. The proportion of mothers with some college education or higher ranged between 18.2% in Portugal and 87.1% in Canada, while the percentages among fathers ranged from a low of 14.3% in Portugal to a high of 83.4% in Canada. The Kenyan site, having the lowest proportion of children that were overweight also had the highest proportion of mothers classified as overweight (67.3%), while China, with one of the highest proportions of childhood overweight, had the lowest proportion of mothers classified as overweight (25.7%). The lowest proportion of overweight among fathers was in China (48.7%), while the highest was in the USA at 81.7%.

**Table 1:** Site-specific descriptive characteristics.

			Child Variables				Parental Variables			
Country (Site)	World Bank Ranking	Analytic Sample*(n)*	Boys *n*(%)	Child Ow *n*(%)	Daily MVPA Minutes mean (SD)	Met MVPA Guidelines *n*(%)	Maternal Some college or higher *n*(%)	Paternal Some college or higher *n*(%)	Maternal Ow *n*(%)	Paternal Ow *n*(%)
Australia (Adelaide)	H	**377**	183 (48.5)	139 (36.9)	65.3 (23.2)	207 (54.9)	274 (72.7)	252 (66.8)	185 (49.1)	262 (70.0)
Brazil (São Paulo)	U-M	**342**	168 (49.1)	152 (44.4)	58.6 (24.7)	143 (41.8)	99 (29.0)	96 (28.1)	174 (50.9)	245 (71.6)
Canada (Ottawa)	H	**464**	191 (41.2)	129 (27.8)	58.8 (19.5)	204 (44.0)	404 (87.1)	387 (83.4)	164 (35.3)	286 (61.6)
China (Tianjin)	U-M	**464**	248 (53.4)	196 (42.2)	45.4 (15.9)	75 (16.2)	196 (42.2)	204 (44.0)	119 (25.7)	226 (48.7)
Colombia (Bogotá)	U-M	**573**	295 (51.5)	142 (24.8)	66.6 (24.0)	326 (56.9)	185 (32.3)	147 (25.7)	207 (36.1)	303 (52.9)
Finland (Helsinki, Espoo & Vantaa)	H	**425**	203 (47.8)	102 (24.0)	71.0 (26.6)	272 (64.0)	276 (64.9)	232 (54.6)	159 (37.4)	257 (60.5)
India (Bangalore)	L-M	**460**	208 (45.2)	151 (32.8)	48.4 (21.0)	116 (25.2)	336 (73.0)	368 (80.0)	222 (48.3)	231 (50.2)
Kenya (Nairobi)	L	**303**	140 (46.2)	57 (18.8)	71.2 (31.9)	167 (55.1)	179 (59.1)	191 (63.0)	204 (67.3)	172 (56.8)
Portugal (Porto)	H	**537**	236 (44.0)	252 (46.9)	55.6 (21.5)	186 (34.6)	98 (18.2)	77 (14.3)	220 (41.0)	338 (62.9)
South Africa (Cape Town)	U-M	**134**	65 (48.5)	41 (30.6)	56.6 (23.4)	48 (35.8)	52 (38.8)	55 (41.0)	80 (59.7)	100 (74.6)
UK (Bath & N.E. Somerset)	H	**328**	144 (43.9)	78 (23.8)	64.4 (22.2)	173 (52.7)	210 (64.0)	193 (58.8)	131 (39.9)	209 (63.7)
US (Baton Rouge)	H	**345**	131 (38.0)	126 (36.5)	49.9 (18.4)	90 (26.1)	274 (79.4)	235 (68.1)	178 (51.6)	282 (81.7)

**Acronyms:** N.E. Somerset (North East Somerset); Ow (Overweight); MVPA (Moderate-to-Vigorous Physical Activity); SD (Standard Deviation). **NOTES: World Bank Ranking**: L (Low Income); L-M (Lower-Middle Income); U-M (Upper-Middle Income); and H (High Income) (27). **Child Ow**: Children categorised as overweight (includes obese). **Daily MVPA Minutes:** Defined as ≥ 574 counts per 15 seconds (31). **Met MVPA Guidelines:** The proportion of children meeting the WHO MVPA guidelines of ≥60 min/day (8). **Maternal or Paternal Education**: The proportions represented in the table include parents reporting attaining some college/associate degree, bachelor, or post-graduate degree, compared to the remainder reporting that they completed high school, have some high school, or less than high school. **Maternal or Paternal Ow:** Parents categorised as overweight (includes those classified as obese).

Parental factors associated with childhood overweight are presented in *Table 2*. Results showed that maternal education was positively associated with childhood overweight for the Colombian and Kenyan sites. Children from Colombia and Kenya had a 1.9 and 4.8 times higher odds of being overweight respectively, if their mothers had attained some college education or higher compared to children with mothers who had a lower education level. Similarly, Kenyan children had a 5.0 times higher odds of being overweight if their fathers had a higher education level. In contrast, children from Brazil and the USA had 45% and 46% lower odds respectively of being overweight if their fathers had attained a higher education level. For all sites besides Kenya, maternal and child overweight were positively associated, with children at a 1.5 to 2.9 times higher odds of being overweight if their mothers were overweight. The association (odds ratio [95% confidence interval]) between child and father overweight was also positive for the Brazil (2.0 [1.2–3.3]), China (3.2 [2.1–4.7]), Colombia (2.2 [1.5–3.3]), Finland (1.6 [1.0–2.7]), India (2.4 [1.6–3.7]), Kenya (3.5 [1.7–7.2]), and Portugal (1.4 [1.0–2.1]) sites.

**Table 2:** Parental factors associated with child overweight.

			Maternal Education		Paternal Education		Maternal Ow		Paternal Ow	
Country (Site)	World Bank Ranking	Analytic Sample *(n)*	OR	95% CI	OR	95% CI	OR	95% CI	OR	95% CI
Australia (Adelaide)	H	**377**	1.12	0.68–1.83	0.80	0.50–1.27	2.60	1.66–4.05*	1.36	0.84–2.20
Brazil (São Paulo)	U-M	**342**	0.78	0.47–1.28	0.55	0.33–0.92*	2.40	1.53–3.76*	2.01	1.20–3.34*
Canada (Ottawa)	H	**464**	0.80	0.44–1.47	0.70	0.41–1.20	2.36	1.53–3.63*	1.35	0.87–2.10
China (Tianjin)	U-M	**464**	1.33	0.87–2.04	0.91	0.59–1.39	1.55	1.01–2.39*	3.17	2.13–4.70*
Colombia (Bogotá)	U-M	**573**	1.90	1.23–2.94*	1.51	0.95–2.40	2.05	1.38–3.05*	2.20	1.46–3.31*
Finland (Helsinki, Espoo & Vantaa)	H	**425**	0.93	0.57–1.50	0.66	0.42–1.06	2.78	1.75–4.43*	1.65	1.01–2.69*
India (Bangalore)	L-M	**460**	1.49	0.92–2.41	1.50	0.87–2.60	2.16	1.44–3.25*	2.43	1.60–3.69*
Kenya (Nairobi)	L	**303**	4.80	2.21–10.43*	5.00	2.03–12.33*	1.37	0.71–2.64	3.53	1.73–7.20*
Portugal (Porto)	H	**537**	1.57	0.98–2.50	0.72	0.42–1.21	1.52	1.06–2.16*	1.45	1.01–2.08*
South Africa (Cape Town)	U-M	**134**	0.90	0.41–2.01	1.49	0.69–3.26	2.22	0.98–5.03	1.23	0.50–3.01
UK (Bath & N.E. Somerset)	H	**328**	0.89	0.51–1.53	0.65	0.38–1.11	2.34	1.38–3.96*	1.54	0.87–2.73
USA (Baton Rouge)	H	**345**	0.59	0.34–1.04	0.54	0.33–0.88*	2.92	1.80–4.72*	1.65	0.87–3.11

**Acronyms:** OR (Odds Ratios); CI (Confidence Interval); N.E. Somerset (North East Somerset); Ow (Overweight).

* Indicates significance at p<0.05.

*Table 3* presents parental factors associated with children meeting the MVPA guidelines. Children from Colombia, Kenya, and Portugal had 47%, 65% and 46% lower odds of meeting MVPA guidelines respectively, if their mothers had some college education or higher compared to a lower education level. Children in Kenya and South Africa also had 73% and 63% lower odds of meeting MVPA guidelines if their fathers had a higher education level. Paternal (but not maternal) overweight was negatively associated with children meeting MVPA guidelines in Kenya, where children had 53% lower odds of meeting MVPA guidelines if their fathers were overweight.

**Table 3:** Parental factors associated with child meeting WHO MVPA guidelines.

			Maternal Education		Paternal Education		Maternal Ow			Paternal Ow	
Country (Site)	World Bank Ranking	Analytic Sample *(n)*	OR	95% CI	OR	95% CI	OR	95% CI		OR	95% CI
Australia (Adelaide)	H	**377**	1.18	0.70–1.99	1.57	0.96–2.56	0.92		0.58–1.45	0.94	0.57–1.53
Brazil (São Paulo)	U-M	**342**	0.74	0.43–1.28	0.64	0.37–1.11	0.75		0.47–1.21	0.67	0.40–1.13
Canada (Ottawa)	H	**464**	1.41	0.75–2.62	1.15	0.66–2.00	0.79		0.51–1.23	0.76	0.50–1.15
China (Tianjin)	U-M	**464**	1.05	0.57–1.94	0.76	0.40–1.43	0.79		0.42–1.47	0.88	0.52–1.49
Colombia (Bogotá)	U-M	**573**	0.53	0.33–0.85*	0.65	0.39–1.09	1.01		0.69–1.48	0.70	0.49–1.01
Finland (Helsinki, Espoo & Vantaa)	H	**425**	1.26	0.79–2.02	1.05	0.67–1.66	1.01		0.65–1.59	0.70	0.45–1.09
India (Bangalore)	L-M	**460**	0.99	0.55–1.76	0.96	0.51–1.78	1.12		0.70–1.81	0.95	0.59–1.52
Kenya (Nairobi)	L	**303**	0.35	0.19–0.63*	0.27	0.14–0.50*	1.04		0.60–1.80	0.47	0.28–0.81*
Portugal (Porto)	H	**537**	0.54	0.31–0.96*	0.66	0.36–1.20	0.95		0.63–1.42	1.35	0.90–2.20
South Africa (Cape Town)	U-M	**134**	0.69	0.28–1.71	0.37	0.15–0.90*	0.67		0.30–1.49	0.55	0.23–1.36
UK (Bath & N.E. Somerset)	H	**328**	0.78	0.47–1.30	1.02	0.62–1.68	0.65		0.40–1.05	0.79	0.48–1.30
USA (Baton Rouge)	H	**345**	1.08	0.52–2.25	1.48	0.79–2.79	0.85		0.49–1.48	1.18	0.59–2.33

**Acronyms:** WHO MVPA (World Health Organization—Moderate-to-vigorous intensity physical activity); OR (Odds Ratios); CI (Confidence Interval); N.E. Somerset (North East Somerset); Ow (Overweight or Obese).

* Indicates significance at p<0.05.

**Fig 1:**
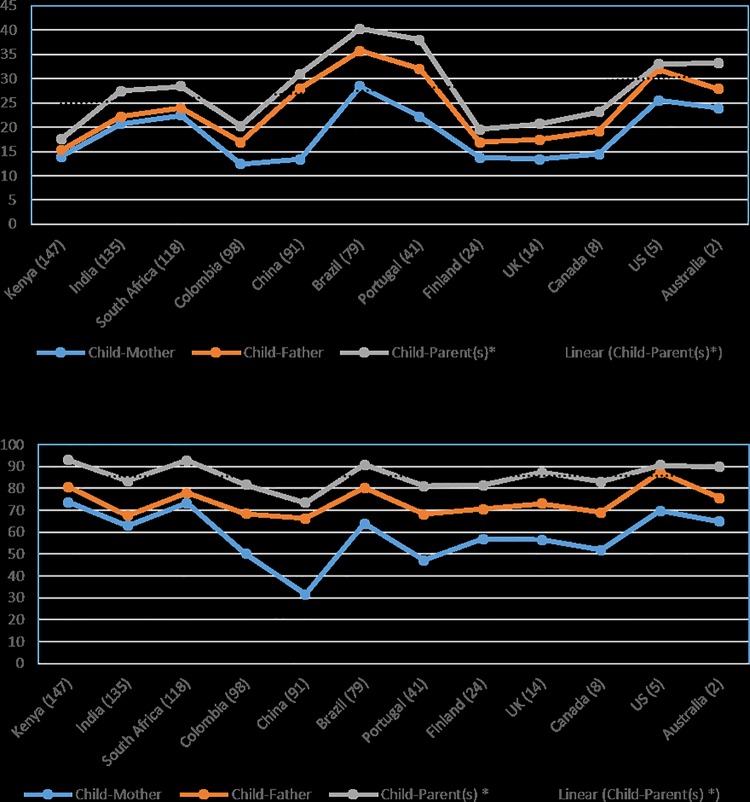
Household coexistence of parental and child overweight. The number beside each country represents its World Bank Human Development Index (HDI) rating (2013).

In order to further explore the relationship between parental and childhood overweight, the coexistence of overweight in the same household was investigated (*Table 4* and *Fig 1*). In the total sample of children, the range of child-parent overweight coexistence was between 17.5% in Kenya and 40.3% in Brazil. Among overweight children, the range of child-parent overweight coexistence was between 73.5% in China and 93.0% in Kenya. *Fig 1* shows the household coexistence of parent-child overweight in the total analytic sample, and in overweight children, with countries ordered by Human Development Index (HDI) status. There were higher proportions of household parent-child overweight coexistence among overweight children in all countries, but no apparent coexistence trend in moving from lower to higher economic status countries.

**Table 4:** Household coexistence of parental and child overweight.

		Proportion in Total Sample (*n* = 4752)			Proportion among Overweight Children (*n* = 1565)		
Country (Site)	Rank	Child-Mother	Child-Father	Child-Parent(s) ^†^	Child-Mother	Child-Father	Child-Parent(s) ^†^
**Overall Sample**		18.2*	23.9*	27.7*	55.1*	72.5*	84.1*
Australia (Adelaide)	H	23.9*	27.9	33.2*	64.8*	75.5	89.9*
Brazil (São Paulo)	U-M	28.4*	35.7*	40.3*	63.8*	80.3*	90.8*
Canada (Ottawa)	H	14.4*	19.2*	23.1*	51.9*	69.0*	83.0*
China (Tianjin)	U-M	13.4*	28.0*	31.0*	31.6*	66.3*	73.5*
Colombia (Bogotá)	U-M	12.4*	16.9*	20.2*	50.0*	68.3*	81.7*
Finland (Helsinki, Espoo & Vantaa)	H	13.7*	16.9*	19.5*	56.9*	70.6*	81.4*
India (Bangalore)	L-M	20.7*	22.2*	27.4*	62.9*	67.6*	83.4*
Kenya (Nairobi)	L	13.9	15.2*	17.5*	73.7	80.7*	93.0*
Portugal (Porto)	H	22.2*	32.0*	38.0*	47.2*	68.2*	81.0*
South Africa (Cape Town)	U-M	22.4*	23.9	28.4	73.2*	78.0	92.7
UK (Bath & N.E. Somerset)	H	13.4*	17.4	20.7*	56.4*	73.1	87.2*
US (Baton Rouge)	H	25.5*	31.9*	33.0	69.8*	87.3*	90.5

^**†**^ Examines the level of overweight coexistence between the child and at least one parent in the household.

* Indicates significance at p<0.05 for Fisher’s exact test for household coexistence of overweight.

## Discussion

### Parental weight status and child overweight

Maternal and child overweight were positively related in all sites except Kenya. A similar positive association between paternal and child overweight was found for seven of the twelve sites (Brazil, China, Colombia, Finland, India, Kenya, and Portugal). These results are aligned with a number of past studies showing that parental overweight, and perhaps more predominantly maternal overweight status, is associated with a higher likelihood of childhood overweight [10–18]. Similarly, our findings showed that maternal overweight was more consistently (in more of the sites) associated with child overweight compared to paternal overweight. Further, and as previously determined, the relationship between parental and child overweight status was fairly consistent across the different sites given our older age group of children 9–11 years [10–12, 15].

### Parental education and child overweight

Children from the Colombian and Kenyan sites were more overweight if their mothers had attained a higher education level (positive association). Kenyan children were also more overweight if their fathers had a higher education level. In contrast, children from the Brazil and USA sites were less overweight if their fathers had attained some college education or higher (negative association). While studies conducted in other lower economic status countries like Colombia and Kenya have reported a similar positive association between parental education level and child BMI [9, 19, 22], results from wealthier countries such as Brazil and the USA have reported a negative relationship between these factors [20, 21]. We speculate that these findings may be potentially explained by a higher level of awareness and knowledge among the more educated parents in higher economic status countries in regard to the positive effects of maintaining a healthy body weight. Having been exposed to an obesogenic environment earlier on [1], strategies to prevent overweight may be better established in these countries. Our findings further seem to indicate that the positive influence of parental higher education on child overweight in higher economic status countries is stronger with fathers than with mothers. In contrast, in lower economies, higher parental education is associated with a higher likelihood of their children being overweight [22]. This may be a consequence of social norms, whereby, in some developing countries, a more overweight child may be perceived as a "healthy child", with adequate food and food security aspired to, particularly for families with more disposable income. In such families, children may also have access to more motorised transport, and engage in less active travel [33]. This, coupled with minimal knowledge about the associated health risks of being overweight, may explain the positive association between parental education level and child BMI in lower economic status countries. These contrasting findings among some lower and higher income countries provides further evidence of the varying status of a physical activity and nutritional transition occurring in these countries [34, 35].

### Parental weight status and child MVPA

Paternal overweight was negatively associated with Kenyan children meeting the MVPA guidelines. This finding is supported by systematic review evidence from sub-Saharan African countries showing a negative relationship between parental BMI and child physical activity measures [23]. However, it is important to note the limitations inherent in self-reported height and weight among adults and the need for directly measured data to confirm these findings.

### Parental education and child MVPA

The results also showed that children from Colombia, Kenya, and Portugal had lower odds of meeting MVPA guidelines if their mothers had attained a higher education level. Kenyan and South African children were also at a lower odds of meeting MVPA guidelines if their fathers had some college education or higher. Fewer studies have investigated the relationship between parental education level and child physical activity; however, evidence points to a negative association between parental education and child physical activity in lower economic status countries [9, 23], and a positive relationship between these factors in higher economic status countries [24], a pattern which is supported by the present study. It is noteworthy that mothers’ education level is more often examined and relationships consistently found in these papers [9, 24, 25]. Our results seem to indicate that the negative influence of higher maternal education on child physical activity is stronger than higher paternal education level in lower economic status countries. We speculate that this may be the result of mothers with higher education in lower economic status countries, having greater access to motorized transport as a consequence of greater SES, and being primarily responsible for the transport means used by their children.

### Household coexistence of parent-child overweight

Interestingly, we found that Kenya had the lowest proportion of overweight children, the highest proportion of overweight mothers, and the highest proportion of child-parent (and child-mother) overweight dyads in the same household compared to other sites. China had the highest proportion of overweight children, the lowest proportion of overweight mothers, and the lowest proportion of child-parent (including both child-mother and child-father) overweight dyads. The highest proportion of overweight among fathers was in the USA, which also had the highest proportion of child-father overweight compared to other sites. These findings reinforce the significant relationships between both mother and father overweight and child overweight. The seemingly higher proportions of child-parent overweight coexistence in middle and high economic status countries in the total sample of children may be a result of longer lasting (over time) pathways of familial susceptibility among children and their parents in higher economic status countries. Familial aggregation has also been shown with physical activity [36]. Since many higher economic status countries have experienced an obesogenic environment for longer periods than low- and middle-income countries [1], it is reasonable to expect higher levels of child-parent overweight coexistence in the same household; however, the inverse parental education-child overweight relationship in higher income countries suggests a conflicting paradigm.

There are a few notable limitations to these analyses including the cross-sectional design with all of its intrinsic weaknesses. Mothers or fathers proxy-reporting for the other parent, or guardians proxy-reporting for biological parents of the child, may also reduce accuracy of results. Excluding children with missing data on any of our variables of interest in the analyses reduced the sample size as previously discussed; however, Fisher’s exact tests were performed to ascertain that excluded children were not statistically different from included children. In addition, the influence of differing social or cultural norms between countries was not captured in this study, for instance, overweight could be a desired trait in some countries [37]. Nonetheless, since parental overweight and education (among other measures of SES) are related to child overweight and MVPA in different ways in developing verses developed countries (countries in different developmental stages), ISCOLE presented an ideal opportunity to examine these relationships.

## Conclusion

With the alarming increases in the prevalence of childhood overweight in some parts of the world, and generally declining physical activity levels, it is important to investigate risk factors and correlates associated with these outcomes in children, which could better inform public health efforts to curb this growing threat. Using data collected in 12 countries, based on a standardised study design, this paper explored the relationships between reported parental education level or overweight, and directly measured childhood overweight and physical activity. The results revealed positive relationships between parental and child overweight, with maternal overweight more consistently associated with child overweight compared to paternal overweight. The results also point to a more positive influence of paternal rather than maternal higher education on lowered child overweight in higher economic status countries, and a more negative influence of maternal higher education on increased child overweight in lower economic status countries. We found negative relationships between parental education and child physical activity in Colombia, Kenya, Portugal, and South Africa. Taken together, the findings indicate that these relationships are related to the developmental stage of different countries; however, to determine the existence of linear (or other) relationships between parental and child factors and the economic or developmental stage of countries, data from more countries of varying status would be required. We speculate that these between country differences may be a consequence of social norms and perceptions that a healthy child should be rounder, with more educated parents moving up in social status and preferring motorised transport and richer foods, coupled with a lower understanding of the importance of maintaining healthy body weights and participating in adequate levels of physical activity among parents from lower rather than higher economic status countries. Though this was not directly measured by ISCOLE, this may represent an important point of intervention for public health administrators and other pertinent stakeholders. This study also underscores the importance of exploring the family context in investigation of child overweight and meeting physical activity guidelines.
